# Occupational Contact Urticaria, Protein Contact Dermatitis and Concomitant Airway Diseases in the Finnish Register of Occupational Diseases in 2005–2020: Main Causes and Occupations at Risk

**DOI:** 10.1111/cod.70137

**Published:** 2026-03-12

**Authors:** Ville Ojanen, Liisa Airaksinen, Hille Suojalehto, Kirsi Koskela, Maria Pesonen

**Affiliations:** ^1^ Occupational Medicine Finnish Institute of Occupational Health Tampere Finland; ^2^ Occupational Medicine Finnish Institute of Occupational Health Helsinki Finland

**Keywords:** contact urticaria, occupational asthma, occupational rhinitis, protein contact dermatitis, registry study

## Abstract

**Background:**

Occupational contact urticaria (OCU) and protein contact dermatitis (PCD) may co‐exist with occupational asthma (OA) and occupational rhinitis (OR) and be caused by the same allergen. These diseases are commonly caused by immediate (immunoglobulin E (IgE)‐mediated) allergic sensitisation. Limited data exist on the prevalence and causes of concomitant occupational skin and airway diseases (CSAD).

**Objectives:**

To analyse data from the Finnish Register of Occupational Diseases (FROD) for prevalence, main causes, and at‐risk occupations for concomitant occupational skin and airway diseases.

**Methods:**

Data on recognised cases of OCU, PCD, OA and OR in the years 2005–2020 (*n* = 2413) were retrieved from FROD and analysed.

**Results:**

Among OCU/PCD cases, 27% had OA, OR, or both. Conversely, 10% of OA cases and 14% of OR cases had OCU/PCD. Concomitant diseases were most common in farming, animal care, and food‐related jobs. Shared causative exposures were identified in 89% of cases, with cow dander, wheat flour, and chemicals being the most frequent.

**Conclusions:**

Concomitant occupational skin and airway diseases caused by shared work‐related allergens are relatively common. When evaluating patients with occupational immediate allergy, both skin and airway symptoms should be considered.

## Graphical abstract

This Finnish registry study shows that concomitant occupational asthma and rhinitis are common in patients with occupational contact urticaria or protein contact dermatitis, often sharing the same exposure. When evaluating occupational immediate allergy, both skin and airway symptoms should be considered. Interdisciplinary cooperation is essential for diagnosing concomitant occupational diseases.

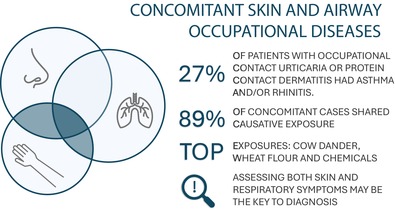

## 1 Introduction

Although immediate (immunoglobulin E (IgE)‐mediated) allergic sensitisation may cause both cutaneous and airway diseases, previous literature on the prevalence and causes of concomitant occupational immediate skin and airway diseases is relatively limited [1, 2, 3, 4]. Occupational and nonoccupational simultaneous immediate skin and airway diseases have mostly been described in case reports.

Contact urticaria (CU) is defined as a wheal and possibly also flare reaction of the skin, which appears immediately after contact with an eliciting substance and usually clears within a few hours [5].

Protein contact dermatitis (PCD) is an allergic skin reaction described as recurrent, itchy, sometimes vesicular eczema on the contact site of the causative agent [6, 7]. The mechanism of PCD is not known, but in occupational settings, PCD may be diagnosed when immediate allergy to a proteinaceous material is associated with eczema on the contact site. PCD may be accompanied by an initial wheal reaction consistent with CU. CU and PCD are usually caused by immediate allergies, which also carry a risk of development of allergic asthma and allergic rhinitis.

Previous study indicates that concomitant airway diseases are frequent among patients with occupational CU (OCU) or PCD. Of patients diagnosed with OCU or PCD, 46% had either occupational asthma (OA) or occupational rhinitis (OR) caused by the same agent as the skin disease [2].

Sensitiser‐induced OA or OR is induced by a specific workplace sensitiser through a mechanism that is associated with a specific immunological response [8]. Causal agents are commonly protein allergens that can cause the production of specific IgE antibodies. However, chemicals and metals commonly cause OA and OR via IgE‐independent mechanisms. In Finland, diagnosis of sensitiser‐induced OA is based on diagnosis of asthma, detection of specific IgE to an occupational agent and positive peak flow monitoring at work and off work, or positive specific inhalation challenge [9, 10, 11]. OR diagnosis is made in patients with work‐related rhinitis symptoms, detection of immediate sensitisation if relevant, and confirmation with a nasal challenge test or inhalation challenge test.

To provide data on prevalence, main causes and occupations at risk of concomitant occupational immediate skin and airway diseases, we analysed data on such cases recorded in the Finnish Register of Occupational Diseases (FROD) in the years 2005–2020.

For brevity, we use the term ‘skin disease’ for OCU/PCD, ‘airway disease’ for OA, OR and the combination of OA and OR, and ‘concomitant skin and airway disease’ (CSAD) for the combination of CU/PCD, and OA or OR or both.

## 2 Materials and Methods

According to the legislation, the national reimbursement system for occupational diseases in Finland is based on insurances [12]. Statutory accident insurance for occupational accidents and diseases covers all employees, self‐employed farmers, and other categories such as trainees or students. Self‐employed entrepreneurs other than the compulsorily insured farmers may obtain the insurance voluntarily. Consequently, private entrepreneurs who do not opt for voluntary insurance against occupational accidents and diseases may not have their occupational diseases recognised.

An occupational disease is defined in Finland as a medical condition primarily induced by exposure to physical factors, chemical substances, or biological agents encountered during work. The causal relationship between disease and exposure is established when the exposure factor is present in the work environment to a degree sufficient to predominantly cause the disease. In cases of occupational allergies, sensitisation is identified at the individual level. Rigorous differential diagnostics are required.

As explained elsewhere [7], the Finnish Register of Occupational Diseases (FROD) is maintained by the Finnish Institute of Occupational Health (FIOH). The annual data on suspected and recognised occupational diseases are provided by a coordinator of the Finnish insurance institutions, Finnish Workers' Compensation Center, that collects data from all other private insurance companies except the Farmers' Social Insurance Institution which provides the data independently. In addition, the FROD also receives copies of physicians' occupational disease notifications to Regional State Administrative Agencies. Although statutory, these reports cover only a minority of cases and therefore, they provide some complementary information to the register. One case may have up to three concomitant diagnoses and three causative agents registered. The FROD also includes information on the recognition date of the occupational disease, occupation and branch of industry, as well as demographic data including age at recognition and sex.

Occupations are coded according to the Finnish Classification of Occupations 2010 (based on ISCO‐08 classification) which has been in use since 2011. The preceding version of the national classification (Finnish Classification of Occupations 2001 based on ISCO‐88 classification) was used during 2005–2010. The occupational categories of occupational disease cases from 2005–2010 were converted to the latest occupational classification before data analysis. In the register data, occupations are generally classified at the four‐digit level; however, agricultural entrepreneurs are mostly classified at the two‐digit level. In the present study, we have used the most accurate level of occupation code available for each case.

We have limited the present analyses to recognised cases of OCU, PCD, OA and OR. As cases with OCU and PCD cannot be reliably separated from each other in the FROD data, we have analysed them as one group. OA and OR groups comprised of sensitiser‐induced OA and OR cases, and cases caused by irritant agents and those related to moisture damaged buildings were omitted from the analysis. As the aim of the study was to explore and describe occupational CSAD, we combined OA and OR in the results as airway diseases when appropriate.

For the purposes of this study, we retrieved from the register data in the years 2005–2020 of all recognised cases of OCU and PCD, OA or OR. To calculate incidences in various occupations, we utilised the mean workforce in those occupations in years 2010–2020 provided by Statistics Finland [13]. The recognition dates of occupational airway and skin diseases were analysed to estimate the order of appearance of the diseases in cases with CSAD. According to the existing rules on publishing from FROD to maintain the privacy of individuals, we have omitted details of subgroups with less than three cases. The study was conducted in accordance with the ethical principles of the Declaration of Helsinki. Ethical approval was not required for this study as it used administrative register‐based data.

For incidences across occupations, exact 95% confidence intervals were calculated using the Poisson distribution in Microsoft Excel (Microsoft Corp., Redmond, WA, USA). Differences in sex distribution were analysed using Pearson's chi‐square test in SPSS Statistics, version 30.0 (IBM Corp., Armonk, NY, USA).

## 3 Results

### 3.1 Occurrence of Occupational Immediate Skin and Airway Diseases and Concomitant Diseases

**FIGURE 1:**
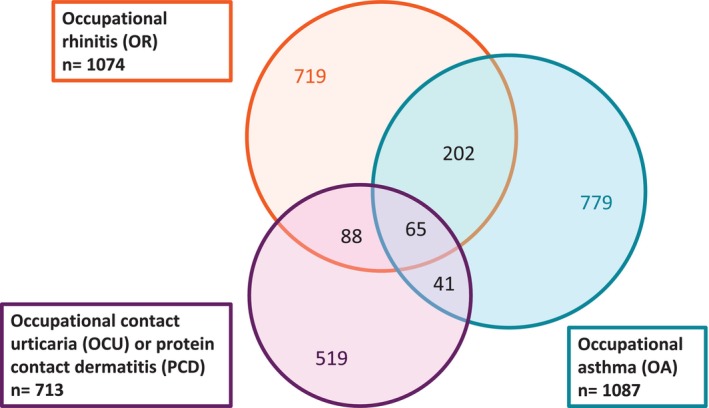
Diagnoses and concomitant diagnoses in cases diagnosed with occupational airway and skin diseases (occupational rhinitis, occupational asthma, occupational contact urticaria and/or protein contact dermatitis) in 2005–2020 according to the Finnish Register of Occupational Diseases.

We investigated a total of 2413 cases diagnosed with OCU/PCD, OA, OR, or a combination of these. OCU/PCD was diagnosed in a total of 713 cases, OA in 1087, and OR in 1074 cases (Figure 1). Any combination of the diseases (more than one of the diseases diagnosed) occurred in 396 cases (16%). Of the total of 1894 cases with airway disease, 202 (11%) had a combination of OA and OR. CSAD was seen in 194 (8%) cases. The co‐occurrence of diseases was documented in 10% of airway disease cases with skin disease and in 27% of skin disease cases with airway involvement. Of the 713 cases with skin disease, 153 (21%) also had concomitant OR, and 106 (10%) had concomitant OA. Conversely, concomitant OCU/PCD occurred in 10% of cases with OA and 14% of cases with OR. The combination of all three diagnoses, namely OCU/PCD, OA, and OR, was seen in 65 (3%) cases. In the majority (75%) of cases with CSAD, both occupational diseases were recognised within the same calendar year (Table 1).

**TABLE 1:** Order of recognition of occupational airway and skin diseases according to the year of recognition of the first airway disease (occupational asthma or occupational rhinitis) as compared to the year of recognition of the skin disease (occupational contact urticaria or protein contact dermatitis) in cases with concomitant occupational skin and airway disease (CSAD).

Timing of recognition of the airway disease as compared to the skin disease	*n*	% of total
≥ 3 years before	8	4.1
2 years before	5	2.6
1 year before	9	4.6
Same year	146	75
1 year after	13	6.7
2 years after	9	4.6
≥ 3 years after	4	2.1
Total	194	100

### 3.2 Demographic Factors

**FIGURE 2:**
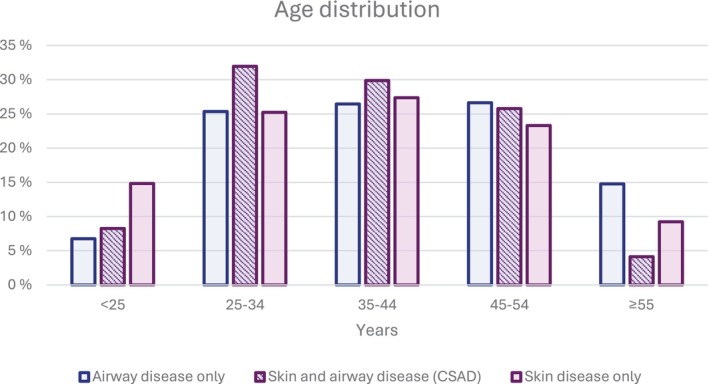
Age distribution of cases with airway disease only, concomitant skin and airway disease (CSAD), and skin disease only. Airway disease only, occupational asthma and/or occupational rhinitis; CSAD, concomitant contact urticaria and/or protein contact dermatitis and occupational asthma and/or occupational rhinitis; Skin disease only, occupational contact urticaria and/or protein contact dermatitis.

The distribution of age at the time of first diagnosis is presented in Figure 2. The mean age of cases with skin disease only was 39, 42 years in those with airway disease only, and 39 years in those with CSAD.

**FIGURE 3:**
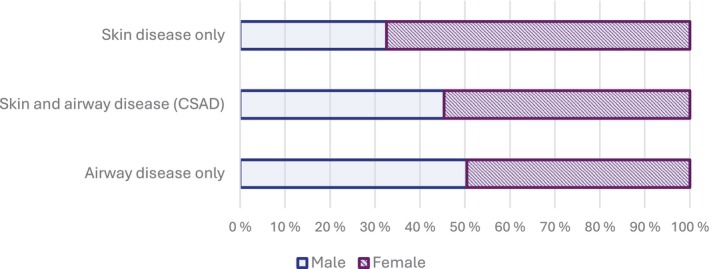
Sex distribution among cases with skin disease only, concomitant skin and airway disease (CSAD), and those with airway disease only. Airway disease only, occupational asthma and/or occupational rhinitis; CSAD, concomitant contact urticaria and/or protein contact dermatitis and occupational asthma and/or occupational rhinitis; Skin disease only, occupational contact urticaria and/or protein contact dermatitis.

Of the total of 2413 cases, 1299 (54%) were women. Airway disease only was equally common in men and women, whereas skin disease only was diagnosed more often in women than in men (Figure 3). Sixty‐four percent of all cases of skin disease, 67% of those with skin disease only, and 55% of those with CSAD were female. The difference in sex distribution between the skin disease only and CSAD groups was statistically significant (*p* = 0.002), whereas no significant difference was observed between the airway disease only and CSAD groups (*p* = 0.183).

### 3.3 Occupations

Occupations with the highest incidence of CSAD were ‘bakers, pastry‐cooks and confectionery makers’ (4.46 per 10 000 person years) followed by ‘mixed crop and animal producers’ i.e., farmers or agricultural workers (3.46), and ‘gardeners, horticultural and nursery growers’ (1.25, Tables 2 and S2). These occupations also had the highest incidences of airway disease only and skin disease only, with clearly higher incidences of airway disease only. Within individual occupations, the share of CSAD was highest in occupations in farming and animal care, 21% in ‘gardeners, horticultural and nursery growers’ and 11% in ‘mixed crop and animal producers’ as compared to other occupations with a share of less than 10% (Table 2). Occupations in farming and animal care include the group of ‘other occupations’ with the share of CSADs as high as 25%. ‘Other occupations’ in farming and animal care includes mostly farmers who have their occupational class registered on two‐digit level.

**TABLE 2:** Occupations of cases with concomitant occupational skin and airway diseases (CSAD) grouped into occupations related to farming and animal care, food‐related, industrial and miscellaneous occupations.

Occupations	*N* (row)	CSADs		Skin disease only		Airway disease only	
		*n* (% of row)	Incidence per 10 000 person years (95% CI)	*n* (% of row)	Incidence per 10 000 person years (95% CI)	*n* (% of row)	Incidence per 10 000 person years (95% CI)
Farming and animal care	954	117 (12)		259 (27)		578 (61)	
Mixed crop and animal producers	631	70 (11)	3.46 (2.69–4.37)	156 (25)	7.71 (6.54–9.02)	405 (64)	20.01 (18.10–22.06)
Gardeners, Horticultural and nursery growers	63	13 (21)	1.25 (0.66–2.14)	16 (25)	1.54 (0.87–2.50)	34 (54)	3.27 (2.26–4.57)
Livestock and dairy producers	205	20 (9.8)	0.62 (0.37–0.96)	59 (29)	1.82 (1.38–2.35)	126 (62)	3.88 (3.23–4.62)
Others (incl. Market‐oriented skilled agricultural workers, Veterinarians, Veterinary technicians and assistants, Pet groomers and animal care workers)	55	14 (25)	ND	28 (51)	ND	13 (24)	ND
Food‐related	531	37 (7.0)		106 (20)		388 (73)	
Bakers, pastry‐cooks and confectionery makers	186	18 (9.7)	4.46 (2.64–7.05)	25 (13)	6.19 (4.00–9.14)	143 (77)	35.4 (29.83–41.71)
Food and related products machine operators	117	5 (4.3)	0.21 (0.06–0.49)	14 (12)	0.58 (0.31–0.98)	98 (84)	4.07 (3.30–4.96)
Cooks	191	11 (5.8)	0.17 (0.08–0.31)	56 (29)	0.87 (0.65–1.13)	124 (65)	1.93 (1.60–2.30)
Others (incl. Butchers, Fishmongers and related food preparers, Fast food preparers, Kitchen helpers)	37	3 (8.1)	ND	11 (30)	ND	23 (62)	ND
Industrial incl. Pelt dressers, Tanners and fellmongers, Life science technicians (excluding medical), Chemical products plant and machine operators, Metal working machine tool setters and operators, Motor vehicle mechanics and repairers, House builders, Electrical Equipment Installers and Repairers	79	13 (16)	ND	6 (7.6)	ND	60 (76)	ND
Miscellaneous	151	14 (9.3)		32 (21)		105 (70)	
Hairdressers	74	3 (4.1)	0.15 (0.03–0.43)	20 (27)	0.98 (0.59–1.51)	51 (69)	2.49 (1.85–3.28)
Shop sales assistants	44	4 (9.0)	0.02 (0.00–0.07)	8 (18)	0.05 (0.02–0.10)	32 (73)	0.19 (0.13–0.28)
Others (incl. Chemical and physical science technicians, Biologists, botanists, zoologists and related professionals, Shop keepers, Shop supervisors, Freight handlers, Personal service workers)	33	7 (21)	ND	4 (12)	ND	22 (67)	ND
Occupation changed^a^	13	13 (100)	ND	ND		ND	
Occupations without concomitant diseases^b^	685	0	ND	116 (17)	0.04 (0.03–0.05)	569 (83)	0.19 (0.17–0.21)
Total		194	0.05 (0.04–0.07)	519	0.14 (0.12–0.16)	1700	0.46 (0.44–0.49)

*Note*: For comparison, *n* and incidence of skin disease only (Skin disease only) and airway diseases only (Airway disease only) in occupations with CSADs are shown. ISCO codes and the mean workforce in 2010–2020 for the occupations are presented in Table S2. Incidences calculated and *n* shown for individual occupations with ≥ 3 cases due to privacy reasons. Mean workforce calculated as the mean of workforce in years 2010–2020 according to workforce data from Statistics Finland [13]. CSADs, concomitant occupational contact urticaria and/or protein contact dermatitis and occupational asthma and/or occupational rhinitis; Skin disease only, occupational contact urticaria and/or protein contact dermatitis; Airway disease only, occupational asthma and/or occupational rhinitis.

Abbreviations: CI, confidence interval; ND, not defined.

^a^ Occupation changed, different occupation reported in association with diagnoses of skin and airway diseases.

^b^ Occupations without concomitant diseases, number of cases of skin and airway diseases in occupations without cases of CSADs.

In 13 (7%) of the cases with CSADs, the recorded occupation was changed between the recognition of their skin and airway diagnoses. In seven of these, the occupations associated were in the same occupational sector. In three cases with changed occupation, data on occupation at the time of the first diagnosis was missing.

### 3.4 Causative Exposures

As shown in Table 3, it was common to have the same reported causative exposure for any combination of the immediate occupational diseases. Shared exposure was especially common among cases with OA and OR (95%), whereas it was present in 89% of cases with CSAD (Table S1).

**TABLE 3:** Causative exposures in cases with concomitant occupational skin and airway disease (CSAD).

Exposure group	Main exposure(s) (share of cases within the exposure group, %)	CSADs *n* (% of cases)	Skin disease only *n* (% of cases)	Airway disease only *n* (% of cases)
Animal dander and excretions	Cow dander (84)	88 (45)	238 (46)	336 (20)
Flour, grain and animal feed	Wheat flour (43)	47 (24)	88 (17)	488 (29)
Chemicals	Isocyanates (19)	6 (3)	31 (6)	242 (14)
	Persulfates (14)			
Animals and animal derived materials, others (non‐food)	Storage mites (94)	3 (2)	< 3	202 (12)
Other (non‐food) plants and plant derived materials	Wood, wood dust (58)	< 3	6 (1)	60 (3)
Plant derived foods, flours excluded	Tomato (21)	10 (5)	19 (4)	33 (2)
	Cucumber (11)			
Natural rubber latex		0	54 (10)	3 (0.2)
Ornamental plants	*Ficus benjamina* (35)	7 (4)	17 (3)	24 (1)
	Decorative and wild plants (33)			
Metals	Stainless steel welding fumes^a^ (55)	< 3	< 3	42 (3)
Animal derived foods	Fish (64)	< 3	35 (7)	3 (0.2)
Enzymes	Lactase (28)	6 (3)	5 (1)	14 (0.8)
Others (Undefined and miscellaneous causes)	Undefined organic materials (44)	< 3	24 (5)	242 (14)
No shared cause		22 (11) ^b^	ND	11 (0.6)^c^
Total *n* of cases (%)		194 (100)	519 (100)	1700 (100)

*Note*: CSAD, concomitant contact urticaria and/or protein contact dermatitis and occupational asthma and/or occupational rhinitis; Skin disease only, occupational contact urticaria and/or protein contact dermatitis; Airway disease only, occupational asthma and/or occupational rhinitis.

^a^ Stainless steel welding fumes include potential respiratory sensitisers nickel and chromium; mechanisms of occupational airway diseases caused by metals are not fully known.

^b^ Different causative exposures reported for skin and airway diseases.

^c^ Different causative exposures reported for occupational asthma and occupational rhinitis.

‘Animal dander and excretions’ were reported as causative exposures in 662 (27%) cases. They caused 45% of the CSADs, 46% of cases of skin disease only, and 20% of cases of airway disease only, respectively (Table 3). Of cases caused by ‘Animal dander and excretions’, as many as 84% were caused by cow dander. ‘Flour, grain and animal feed’ (623 (26%) cases) caused 29% of cases of airway disease only, 25% of CSADs, and 17% of skin diseases only, respectively. In the group of ‘Flour, grain and animal feed’, wheat flour was the main exposure with a share of 43% of the cases. ‘Chemicals’ was the third most reported group of exposures, with isocyanates and persulfates as the main causative exposures, followed by the group ‘Others’ that includes unspecified organic materials, and ‘Animals and animal derived materials’ that consists mainly (94%) of storage mites (Table 3). Natural rubber latex (NRL) proteins caused a total of 57 (2%) cases, 54 of which were skin disease only and three airway disease only. NRL therefore caused 10% of all cases of skin diseases only, but no CSADs. Enzymes caused 25 cases, 24% of them CSADs.

89% of cases caused by ‘Animal dander and excretions’ and 80% of those caused by ‘Animals and animal derived materials’ occurred in farming and animal care (data not shown). The latter group of exposures includes storage mites, which were a common cause of airway diseases in farming and animal care. Of cases caused by ‘Flour, grain and animal feed’, 69% occurred in food‐related occupations and 22% in farming and animal care. Of cases caused by ‘Chemicals’, 46% were from industrial occupations and 45% from occupations grouped as miscellaneous, which includes hairdressers as a large group (Table 2). Seventy‐four percent of cases caused by ‘Animal‐derived foods’ occurred in food‐related occupations whereas the major share of cases caused by ‘Plant‐derived foods’ was divided between food‐related occupations (46%) and plant cultivation (40%). 64% of cases caused by NRL worked in the health care sector.

## 4 Discussion

Based on national register data on occupational immediate skin and airway diseases diagnosed in Finland in 2005–2020, we found CSAD in 8% of all analysed cases. Concomitant airway disease was common in cases with OCU/PCD as 27% of them were also diagnosed with OA, OR, or both. Conversely, 10% of cases with OA and 14% of cases of OR had concomitant OCU/PCD.

CSADs were most frequently reported among workers in farming and animal care and in food‐related occupations. The highest incidence of CSAD was seen in ‘bakers, pastry‐cooks and confectionery makers’ followed by ‘mixed crop and animal producers’. These occupations also had the highest incidences of airway diseases only and skin diseases only. We are not aware of any previously published population‐based data on the incidence of occupational CSADs in various occupations.

CSADs shared the same causative exposures in a majority (89%) of cases. The most frequent causative exposures for all cases were cow dander and wheat flour followed by chemicals. Chemicals caused more frequently airway diseases than skin diseases or concomitant diseases. NRL caused mainly skin diseases, and metals (mostly welding fumes) caused mainly airway diseases.

We found women to be overrepresented among cases with CU/PCD, whereas there was no difference between sexes in cases with airway diseases only. Also, in a previous analysis of FROD data on years 2005–2016, women were dominant among cases diagnosed with OCU/PCD (65% of cases), and in fact, in all the occupational skin diseases analysed [14].

Occupational skin and airway diseases were recognised within the same calendar year in a majority (75%) of cases. However, the recognition date in FROD is based on the decision date at the insurance company and it therefore is not equal to the time of the appearance of symptoms of the disease. Consequently, the recognition dates reported herein do not accurately indicate the order of appearance of immediate skin and airway disease symptoms. They may serve as indicative surrogate markers for the order of appearance. Delay in seeking medical attention is likely to vary from patient to patient, and presenting for examinations is perhaps more often prompted by the appearance of the airway rather than the skin symptoms. Moreover, when a suspected occupational disease is being examined, symptoms of other diseases may come under attention, leading to examinations for those, too, which may result in the diseases being recognised at the same time.

Development of immediate allergy (IgE‐mediated, Type 1 hypersensitivity) against a workplace exposure is considered the main mechanism for OA, OR, OCU and PCD. Allergens in immediate allergy are mostly proteins, but also some chemicals may cause immediate sensitisation. However, some exposures such as the isocyanates and welding fumes may cause OA through non‐IgE‐mediated mechanisms, and in nonimmunological OCU, there is no immediate allergy against the causative substance [3, 15]. Whereas delayed allergy (type IV hypersensitivity), the mechanism of allergic contact dermatitis (ACD), is not related to respiratory symptoms, occupational ACD and airway diseases may occur concomitantly in workers exposed to factors that cause immediate allergy and to delayed sensitisers in their work. Furthermore, some chemicals including the isocyanates and persulfates are known causes for delayed allergy and ACD and for occupational airway diseases through either immediate allergy or IgE‐independent mechanisms that are not fully known.

As immediate allergy is the dominant mechanism for OA, OR, OCU and PCD, these diseases might be expected to occur concomitantly especially in such occupational settings, where there is both skin and airway exposure to proteinaceous materials. Previously, it was reported from a clinic specialised on occupational diseases that among patients diagnosed with OCU or PCD, 21% had concomitant OA and 38% had OR caused by the same agent as the skin disease [2]. In a study on Italian food handlers examined for suspected occupational allergic diseases, CSADs was reported in 13% of cases [16]. In a Spanish study that comprised both occupational and nonoccupational cases of CU and PCD, allergic rhinitis was present in 56% and asthma in 17% of patients with CU and/or PCD [17].

As previously reported, OCU and PCD jointly comprise 11% of all recognised occupational skin diseases among Finnish workers according to the FROD data and represent the third most recorded type of occupational skin disease after occupational irritant and allergic contact dermatitis [14, 18]. Previously reported causes of OCU and PCD include exposure to proteins in organic materials such as foodstuffs of plant and animal origin, NRL, and enzymes, and in case of OCU, also some chemicals [2, 6, 18, 19, 20, 21, 22, 23].

Sensitiser‐induced OA and immediate allergic OR are the most common types of OA and OR in FROD data. The main causative agents are cow dander, flour and grain, and other plant‐derived agents [24]. Storage mites are a relatively commonly reported cause of OA and OR in Finnish patients. In many countries, mites are common environmental allergens; consequently, storage mite allergy is usually not considered predominantly occupational. In Finland, instead, house dust mite allergy is rare, and occupational storage mite sensitisation is considered to cause occupational airway diseases in farm environments [25]. According to FROD, the most common chemical agents causing OA and OR are acrylates, hairdressing chemicals, epoxy resin systems, and isocyanates.

Cow dander has since long been the most frequent cause of OCU in Finland [18]. It is also a known cause of occupational PCD, OA and OR [26, 27, 28, 29, 30, 31]. In a previous analysis, 45% of patients diagnosed with OCU or PCD caused by cow dander had simultaneous OA or OR caused by the same agent [2]. In that study, concomitant OCU and airway diseases were observed in workers with immediate allergy to other animals, mainly laboratory animals. Occupational exposure to laboratory animals has been reported to cause immediate allergy, respiratory symptoms, and skin symptoms consistent with contact urticaria [32].

Immediate allergy to flour is among the most common causes of occupational respiratory diseases, and bakers are a known risk group of OA and OR [15, 33, 34, 35, 36]. A case of concomitant PCD and OA caused by flours in a pastry chef was recently described [37, 38]. Immediate allergy to buckwheat flour has caused OA, OR, and concomitant OCU in bakers, but also in a grocery store worker [39].

Previous literature on causes of immediate CSAD includes mostly case reports and series. A review on occupational allergies caused by foods of animal origin describes the presence of immediate allergic reactions on the skin and respiratory or mucosal symptoms triggered with the same immediate allergen as common [4]. Case reports and a review on occupational seafood allergy present OA and concomitant OCU in workers involved in handling and processing seafood (fish, crustaceans, squid) [40, 41, 42]. In addition, concomitant occupational airway disease and OCU have been reported as caused by a variety of foodstuffs of plant and animal origin such as paprika, carrot, pork meat, *Penicillium camemberti* in dry sausage, and white and green tea blends' dust and to tomato and cucumber plants in greenhouse workers [43, 44, 45, 46, 47, 48].

OCU and PCD with concomitant airway disease (OA and OR) caused by alpha‐amylase used as flour additive, as well as by other industrial enzymes such as cellulase, xylanase, lactase, and papain have been reported [49, 50, 51, 52, 53].

Regarding non‐edible plant materials, ornamental indoor plants such as *Ficus benjamina* , *Spathiphyllum* and *Hedera helix* may cause immediate allergy, airway disease, and concomitant OCU in professional plant keepers and gardeners [54, 55, 56]. A case of a florist with OA, OR, and OCU caused by immediate allergy to tulip and Easter lily has also been described [57]. In hairdressers, henna ( *Lawsonia inermis* ) and indigo ( *Indigofera tinctoria* ) used as hair dyes and hydrolysed wheat protein in hair care products have been reported as causes of concomitant OCU, OA, and OR [58, 59, 60]. A case of occupational immediate allergy to gum arabic (*Acacia senegalia*) with concomitant OCU and OR has been described [61]. Cultivation of *Cannabis sativa* has been associated with risk of OA and OCU [62]. Proteins of NRL are well‐known occupational immediate allergens which have caused OCU associated with OR and OA [2]. Immediate allergy to NRL has been very frequent in healthcare workers in the 1990s [63], and thereafter decreased although it still is relevant [64, 65, 66].

Insects may cause occupational CSADs. *Tribolium confusum* , the confused flour beetle, was reported as the cause of OR, OCU, and asthmatic symptoms in a maintenance worker of a crispbread factory, where the beetle thrived in old flours [67]. Occupational exposure to migratory locusts has caused immediate allergy, OA, OR, and OCU in research laboratory personnel and OCU with respiratory symptoms suggestive of OA and OR in a zookeeper [68, 69]. An outbreak of OCU, OA, and anaphylaxis caused by pea weevil ( *Bruchus pisorum* ) among Spanish workers who handled peas infested by it was recently described [70].

Apart from proteins, also some low molecular weight agents may cause airway diseases and CU. Low molecular weight agents reported as causative of concomitant OCU and airway disease include persulfates, hair dye ingredients, acid anhydrides, piperacillin sodium and some other antibiotics, platinum and palladium salts, nickel, 3‐(bromomethyl)‐2‐chloro‐4‐(methylsulfonyl)‐benzoic acid, diisocyanates and chloramine‐T [2, 71, 72, 73, 74, 75, 76, 77, 78, 79, 80, 81].

At diagnosis of the first allergic occupational disease in a patient, complete cessation of work‐related exposure should be prioritised over mere reduction to prevent the progression to more severe conditions. OR is often seen as a precursor to OA, which can be effectively prevented through timely cessation of exposure [82]. In the worst‐case scenario, immediate allergic reactions may potentially lead to anaphylaxis. Occupational anaphylaxis is considered uncommon [83]. The main causes of it include insect venom, food, drugs, and NRL [84].

Primary prevention of allergic occupational diseases involves comprehensive orientation at the commencement of employment and adherence to work practices that minimise exposure to the lowest feasible level. It is crucial to educate workers on recognising potential allergic symptoms and to encourage prompt contact with occupational health services should symptoms arise.

Awareness of the possibility of CSAD is important for any physician that encounters patients with potential work‐related health problems. In cases of suspected immediate allergic occupational diseases, it is essential to evaluate both skin and respiratory symptoms across all levels of health care and within all medical disciplines. The diagnostic process of occupational skin and airway diseases may occur in specialised health care and be divided between disciplines due to the symptoms arising from different organ systems. The disciplines involved may vary by country but can include dermatology, otorhinolaryngology, pulmonology, allergology, and occupational medicine. Interdisciplinary cooperation, including effective consultation and referral practices, is important for the timely and accurate diagnosis of CSADs.

Diagnosing multiple allergic occupational diseases is particularly important, as individuals with simultaneous diagnoses are significantly more likely to require vocational rehabilitation compared to those with a single diagnosis. Vocational rehabilitation is often necessary to ensure both exposure cessation and career continuity [85].

## 5 Conclusion

According to the Finnish national registry data, immediate occupational CSAD caused by the same exposures at work is relatively common, as it occurred in 8% of all cases of immediate occupational diseases. The main causes include proteins such as cow dander and wheat flour. The occupational groups at risk include farming and animal care and food‐related occupations. When examining patients with suspected occupational diseases caused by immediate allergies, it is important to inquire about both skin and airway symptoms. Simultaneous diagnoses can significantly affect work ability and influence the individual's professional future.

## Author Contributions

**Liisa Airaksinen:** conceptualisation, data curation, writing – review and editing (equal). **Kirsi Koskela:** conceptualisation, writing – review and editing (equal). **Ville Ojanen:** conceptualisation, data curation (lead), formal analysis (lead), writing – original draft (supporting), writing – review and editing (equal), visualisation. **Maria Pesonen:** conceptualisation (lead), data curation, writing – original draft (lead), writing – review and editing (equal). **Hille Suojalehto:** conceptualisation, writing – review and editing (equal).

## Ethics Statement

The study was conducted in accordance with the ethical principles of the Declaration of Helsinki.

## Conflicts of Interest

M.P. has received lecture grants from UCB Pharma Finland in 2023–2024. The other authors declare no conflicts of interest.

## Supporting information


**Table S1:** Proportion of cases with the same causative exposure for combinations of immediate occupational diseases.

**Table S2:** ISCO codes and mean workforces for occupations presented in Table 2. Mean workforce calculated as the mean of workforce in years 2010–2020 according to Statistics Finland [13].

## Data Availability

Dataset analysed in this study is not available.

## References

[1] K. Aalto‐Korte and S. Suomela , “Contact Urticaria Syndrome: Epidemiology and Occupational Relevance,” in Contact Urticaria Syndrome, ed. A. M. Giménez‐Arnau and H. I. Maibach (CRC Press, Taylor & Francis Group, 2015), 13–20.

[2] E. Helaskoski , H. Suojalehto , O. Kuuliala , and K. Aalto‐Korte , “Occupational Contact Urticaria and Protein Contact Dermatitis: Causes and Concomitant Airway Diseases,” Contact Dermatitis 77, no. 6 (2017): 390–396, 10.1111/cod.12856. 28795430

[3] M. Bizjak , O. Aerts , D. Pesque , et al., “Contact Urticaria and Related Conditions: Clinical Review,” Contact Dermatitis 93, no. 2 (2025): 87–107, 10.1111/cod.14794. 40174899 PMC12223959

[4] H. Dickel , “Exceptional Occupational Allergies due to Food of Animal Origin,” Der Hautarzt 72, no. 6 (2021): 493–501, 10.1007/s00105-021-04810-8. 33877379 PMC8169499

[5] A. M. Gimenez‐Arnau , D. Pesque , and H. I. Maibach , “Contact Urticaria Syndrome: A Comprehensive Review,” Current Dermatology Reports 11, no. 4 (2022): 194–201, 10.1007/s13671-022-00379-0. 36415744 PMC9672538

[6] N. Hjorth and J. Roed‐Petersen , “Occupational Protein Contact Dermatitis in Food Handlers,” Contact Dermatitis 2, no. 1 (1976): 28–42, 10.1111/j.1600-0536.1976.tb02975.x. 145923

[7] C. Amaro and A. Goossens , “Immunological Occupational Contact Urticaria and Contact Dermatitis From Proteins: A Review,” Contact Dermatitis 58, no. 2 (2008): 67–75, 10.1111/j.1600-0536.2007.01267.x. 18186738

[8] S. M. Tarlo and C. Lemiere , “Occupational Asthma,” New England Journal of Medicine 370, no. 7 (2014): 640–649, 10.1056/NEJMra1301758. 24521110

[9] H. Suojalehto , V. C. Moore , G. Moscato , P. S. Burge , J.‐L. Malo , and O. Vandenplas , “Functional Assessment,” in Asthma in the Workplace, ed. S. Tarlo , D. I. Bernstein , J.‐L. Malo , and O. Vandenplas (CRC Press, 2022), 89–101.

[10] G. Moscato , O. Vandenplas , R. Gerth Van Wijk , et al., “Occupational Rhinitis,” Allergy 63, no. 8 (2008): 969–980, 10.1111/j.1398-9995.2008.01801.x. 18691299

[11] O. Vandenplas , H. Suojalehto , T. B. Aasen , et al., “Specific Inhalation Challenge in the Diagnosis of Occupational Asthma: Consensus Statement,” European Respiratory Journal 43, no. 6 (2014): 1573–1587, 10.1183/09031936.00180313. 24603815

[12] Finnish Workers' Compensation Center , “Worker's Compensation Act 459/2015,” Unofficial English translation, accessed January 15, 2026, https://www.tyotapaturmatieto.fi/julkaisu/tyotapaturmatietopalvelu/3402?c=25.

[13] Statistics Finland , “Employment: Documentation of Statistics,” 2025 accessed January 15, 2026, https://stat.fi/en/statistics/documentation/tyokay.

[14] K. Aalto‐Korte , K. Koskela , and M. Pesonen , “12‐Year Data on Dermatologic Cases in the Finnish Register of Occupational Diseases I: Distribution of Different Diagnoses and Main Causes of Allergic Contact Dermatitis,” Contact Dermatitis 82, no. 6 (2020): 337–342, 10.1111/cod.13488. 32037572

[15] J. L. Malo and M. Chan‐Yeung , “Agents Causing Occupational Asthma,” Journal of Allergy and Clinical Immunology 123, no. 3 (2009): 545–550, 10.1016/j.jaci.2008.09.010. 18951622

[16] J. Granzotto , I. Lazzarato , M. Mauro , L. Cegolon , and F. Larese Filon , “A 21‐Year Perspective on Occupational Skin and Respiratory Diseases Among Food Handlers,” Medicina del Lavoro 116, no. 4 (2025): 17079, 10.23749/mdl.v116i4.17079. 40762177 PMC12363418

[17] D. Pesqué , P. Torros‐Bosó , E. Andrades , R. M. Pujol , F. Gallardo , and A. M. Gimenez‐Arnau , “Characterization of Contact Urticaria Syndrome Phenotypes: A Retrospective Study of 95 Cases,” Acta Dermato‐Venereologica 105 (2025): adv43837, 10.2340/actadv.v105.43837. 41123357 PMC12560404

[18] M. Pesonen , K. Koskela , and K. Aalto‐Korte , “Contact Urticaria and Protein Contact Dermatitis in the Finnish Register of Occupational Diseases in a Period of 12 Years,” Contact Dermatitis 83, no. 1 (2020): 1–7, 10.1111/cod.13547. 32243591

[19] J. Lukacs , S. Schliemann , and P. Elsner , “Occupational Contact Urticaria Caused by Food ‐ a Systematic Clinical Review,” Contact Dermatitis 75, no. 4 (2016): 195–204, 10.1111/cod.12653. 27425004

[20] L. Vester , J. P. Thyssen , T. Menne , and J. D. Johansen , “Occupational Food‐Related Hand Dermatoses Seen Over a 10‐Year Period,” Contact Dermatitis 66, no. 5 (2012): 264–270, 10.1111/j.1600-0536.2011.02048.x. 22486568

[21] A. Barbaud , C. Poreaux , E. Penven , and J. Waton , “Occupational Protein Contact Dermatitis,” European Journal of Dermatology 25, no. 6 (2015): 527–534, 10.1684/ejd.2015.2593. 26242922

[22] E. Helaskoski , H. Suojalehto , O. Kuuliala , and K. Aalto‐Korte , “Prick Testing With Chemicals in the Diagnosis of Occupational Contact Urticaria and Respiratory Diseases,” Contact Dermatitis 72, no. 1 (2015): 20–32, 10.1111/cod.12308. 25289485

[23] M. Bizjak‐Suran , I. L. Jorgensen , and J. H. Alfonso , “Update on Occupational Contact Urticaria: A Systematic Narrative Review,” Current Opinion in Allergy and Clinical Immunology 26, no. 2 (2026): 84–94, 10.1097/ACI.0000000000001138. 41410133 PMC12955977

[24] K. Koskela , J. Lehtimäki , V. Ojanen , et al., “Ammattitaudit ja ammattitautiepäilyt 2019–2020 Finnish Institute of Occupational Health Helsinki,” 2024, [In Finnish. English summary.], accessed January 15, 2026, https://www.julkari.fi/handle/10024/148219.

[25] H. Suojalehto , M. F. Jeebhay , I. Sander , et al., “Occupational Mite Allergy and Asthma: An EAACI Task Force Report,” Allergy 80, no. 9 (2025): 2484–2500, 10.1111/all.16666. 40799117 PMC12444871

[26] L. Kanerva and P. Susitaival , “Cow Dander: The Most Common Cause of Occupational Contact Urticaria in Finland,” Contact Dermatitis 35, no. 5 (1996): 309–310, 10.1111/j.1600-0536.1996.tb02400.x. 9007384

[27] C. Timmer and P. J. Coenraads , “Allergic Contact Dermatitis From Cow Hair and Dander,” Contact Dermatitis 34, no. 4 (1996): 292–293, 10.1111/j.1600-0536.1996.tb02202.x. 8730169

[28] V. Mahler , T. L. Diepgen , A. Heese , and K. P. Peters , “Protein Contact Dermatitis due to Cow Dander,” Contact Dermatitis 38, no. 1 (1998): 47–48, 10.1111/j.1600-0536.1998.tb05641.x. 9504251

[29] A. L. Valero Santiago , E. Rosell Vives , M. Lluch Perez , J. Sancho Gomez , J. Piulats Xanco , and A. Malet Casajuana , “Occupational Allergy Caused by Cow Dander: Detection and Identification of the Allergenic Fractions,” Allergol Immunopathol (Madr) 25, no. 6 (1997): 259–265. 9469201

[30] M. Rautalahti , E. O. Terho , I. Vohlonen , and K. Husman , “Atopic Sensitization of Dairy Farmers to Work‐Related and Common Allergens,” European Journal of Respiratory Diseases. Supplement 152 (1987): 155–164. 3478214

[31] E. O. Terho , K. Husman , I. Vohlonen , M. Rautalahti , and H. Tukiainen , “Allergy to Storage Mites or Cow Dander as a Cause of Rhinitis Among Finnish Dairy Farmers,” Allergy 40, no. 1 (1985): 23–26, 10.1111/j.1398-9995.1985.tb04150.x. 3977027

[32] G. Agrup , L. Belin , L. Sjostedt , and S. Skerfving , “Allergy to Laboratory Animals in Laboratory Technicians and Animal Keepers,” British Journal of Industrial Medicine 43, no. 3 (1986): 192–198, 10.1136/oem.43.3.192. 3947583 PMC1007632

[33] J. C. McDonald , H. L. Keynes , and S. K. Meredith , “Reported Incidence of Occupational Asthma in the United Kingdom, 1989‐97,” Occupational and Environmental Medicine 57, no. 12 (2000): 823–829, 10.1136/oem.57.12.823. 11077011 PMC1739897

[34] M. Hytonen , L. Kanerva , H. Malmberg , R. Martikainen , P. Mutanen , and J. Toikkanen , “The Risk of Occupational Rhinitis,” International Archives of Occupational and Environmental Health 69, no. 6 (1997): 487–490, 10.1007/s004200050178. 9215937

[35] T. Storaas , S. K. Steinsvag , E. Florvaag , A. Irgens , and T. B. Aasen , “Occupational Rhinitis: Diagnostic Criteria, Relation to Lower Airway Symptoms and IgE Sensitization in Bakery Workers,” Acta Oto‐Laryngologica 125, no. 11 (2005): 1211–1217, 10.1080/00016480510044205. 16353405

[36] O. Vandenplas , V. Doyen , and M. Raulf , “Occupational Respiratory Allergy to Flour in the Modern Era,” Current Allergy and Asthma Reports 25, no. 1 (2025): 22, 10.1007/s11882-025-01204-x. 40289031

[37] S. Alique‐Garcia , J. Company‐Quiroga , E. Gonzalez Mancebo , S. Cordoba Guijarro , A. Garrido‐Rios , and J. Borbujo Martinez , “Protein Contact Dermatitis by Flours in a Pastry Chef,” Contact Dermatitis 80, no. 6 (2019): 403–404, 10.1111/cod.13218. 30653699

[38] V. Doyen , N. Migueres , A. Frère , et al., “Diagnostic Accuracy of Specific IgE Against Wheat and Rye in Flour‐Induced Occupational Asthma,” Journal of Allergy and Clinical Immunology in Practice 12, no. 8 (2024): 2017–2025.e5, 10.1016/j.jaip.2024.05.014. 38768897

[39] S. Jungewelter , L. Airaksinen , and M. Pesonen , “Occupational Buckwheat Allergy as a Cause of Allergic Rhinitis, Asthma, Contact Urticaria and Anaphylaxis‐An Emerging Problem in Food‐Handling Occupations?,” American Journal of Industrial Medicine 63, no. 11 (2020): 1047–1053, 10.1002/ajim.23185. 32944967

[40] M. F. Jeebhay , T. G. Robins , S. B. Lehrer , and A. L. Lopata , “Occupational Seafood Allergy: A Review,” Occupational and Environmental Medicine 58, no. 9 (2001): 553–562, 10.1136/oem.58.9.553. 11511741 PMC1740192

[41] C. Lemiere , A. Desjardins , S. Lehrer , and J. L. Malo , “Occupational Asthma to Lobster and Shrimp,” Allergy 51, no. 4 (1996): 272–273, 10.1111/j.1398-9995.1996.tb04606.x. 8792927

[42] M. Wiszniewska , D. Tymoszuk , A. Pas‐Wyroslak , et al., “Occupational Allergy to Squid ( *Loligo vulgaris* ),” Occupational Medicine (Oxford, England) 63, no. 4 (2013): 298–300, 10.1093/occmed/kqt025. 23535711

[43] S. Jungewelter , L. Airaksinen , and M. Pesonen , “Occupational Rhinitis, Asthma, and Contact Urticaria From IgE‐Mediated Allergy to Pork,” American Journal of Industrial Medicine 62, no. 1 (2019): 80–84, 10.1002/ajim.22921. 30474282

[44] C. Foti , M. Carino , N. Cassano , R. Panebianco , G. A. Vena , and L. Ambrosi , “Occupational Contact Urticaria From paprika,” Contact Dermatitis 37, no. 3 (1997): 135. 9330828

[45] A. Moreno‐Ancillo , A. C. Gil‐Adrados , C. Dominguez‐Noche , P. M. Cosmes , and F. Pineda , “Occupational Asthma due to Carrot in a Cook,” Allergol Immunopathol (Madr) 33, no. 5 (2005): 288–290, 10.1157/13080934. 16287550

[46] R. Merget , I. Sander , P. Rozynek , et al., “Occupational Immunoglobulin E‐Mediated Asthma due to Penicillium Camemberti in a Dry‐Sausage Packer,” Respiration 76, no. 1 (2008): 109–111, 10.1159/000097137. 17108671

[47] H. Suojalehto , P. Holtta , I. Lindstrom , and S. Suomela , “Prevalence of Tomato and Cucumber Sensitization Among Greenhouse Workers,” Journal of Allergy and Clinical Immunology in Practice 10, no. 2 (2022): 640–642, 10.1016/j.jaip.2021.09.038. 34626860

[48] E. Paulsen , L. Hvid , and F. Andersen , “Immediate and Delayed Contact Reactions to White and Green Tea Blends,” Contact Dermatitis 86, no. 2 (2022): 134–136, 10.1111/cod.13992. 34668202

[49] L. Kanerva , M. Vanhanen , and O. Tupasela , “Occupational Allergic Contact Urticaria From Fungal but Not Bacterial Alpha‐Amylase,” Contact Dermatitis 36, no. 6 (1997): 306–307, 10.1111/j.1600-0536.1997.tb00007.x. 9237011

[50] K. Tarvainen , L. Kanerva , O. Tupasela , et al., “Allergy From Cellulase and Xylanase Enzymes,” Clinical & Experimental Allergy 21, no. 5 (1991): 609–615. 1742654 10.1111/j.1365-2222.1991.tb00854.x

[51] L. Kanerva , M. Vanhanen , and O. Tupasela , “Occupational Contact Urticaria From Cellulase Enzyme,” Contact Dermatitis 38, no. 3 (1998): 176–177, 10.1111/j.1600-0536.1998.tb05695.x. 9536420

[52] A. Laukkanen , P. Ruoppi , S. Remes , T. Koistinen , and S. Makinen‐Kiljunen , “Lactase‐Induced Occupational Protein Contact Dermatitis and Allergic Rhinoconjunctivitis,” Contact Dermatitis 57, no. 2 (2007): 89–93, 10.1111/j.1600-0536.2007.01158.x. 17627646

[53] D. Quinones , S. Alonso , R. Lopez , et al., “Contact Urticaria, Rhinoconjunctivitis and Bronchial Asthma From Occupational Use of Papain,” Allergol Immunopathol (Madr) 27, no. 5 (1999): 273–275. 10568879

[54] M. Pesonen and K. Aalto‐Korte , “Occupational Allergic Contact Dermatitis and Contact Urticaria Caused by Indoor Plants in Plant Keepers,” Contact Dermatitis 83, no. 6 (2020): 515–518, 10.1111/cod.13647. 32588436

[55] L. Kanerva , T. Estlander , and K. Aalto‐Korte , “Occupational Protein Contact Dermatitis and Rhinoconjunctivitis Caused by Spathe (Spathiphyllum) Flowers,” Contact Dermatitis 42, no. 6 (2000): 369–370. 10871119

[56] H. Thormann and E. Paulsen , “Contact Urticaria to Common Ivy ( *Hedera helix* cv. ‘Hester’) With Concomitant Immediate Sensitivity to the Labiate Family (Lamiaceae) in a Danish Gardener,” Contact Dermatitis 59, no. 3 (2008): 179–180, 10.1111/j.1600-0536.2008.01389.x. 18759904

[57] P. Piirila , L. Kanerva , K. Alanko , et al., “Occupational IgE‐Mediated Asthma, Rhinoconjunctivitis, and Contact Urticaria Caused by Easter Lily (Lilium Longiflorum) and Tulip,” Allergy 54, no. 3 (1999): 273–277, 10.1034/j.1398-9995.1999.00947.x. 10321564

[58] I. M. Majoie and D. P. Bruynzeel , “Occupational Immediate‐Type Hypersensitivity to Henna in a Hairdresser,” American Journal of Contact Dermatitis 7, no. 1 (1996): 38–40. 8796740 10.1016/s1046-199x(96)90031-7

[59] L. Airaksinen , P. Pallasaho , R. Voutilainen , and M. Pesonen , “Occupational Rhinitis, Asthma, and Contact Urticaria Caused by Hydrolyzed Wheat Protein in Hairdressers,” Annals of Allergy, Asthma & Immunology : Official Publication of the American College of Allergy, Asthma, & Immunology 111, no. 6 (2013): 577–579, 10.1016/j.anai.2013.09.025. 24267377

[60] T. Haltia , S. Jungewelter , L. Airaksinen , S. Suomela , I. Lindstrom , and H. Suojalehto , “Occupational Asthma, Rhinitis, and Contact Urticaria From Indigo ( *Indigofera tinctoria* ) Hair Dye,” Journal of Allergy and Clinical Immunology in Practice 9, no. 9 (2021): 3500–3502, 10.1016/j.jaip.2021.04.047. 33957290

[61] P. Romita , T. Bufano , A. Antelmi , M. Gelardi , and C. Foti , “Occupational Allergic Rhinitis and Contact Urticaria Caused by Gum Arabic in a Candy Factory Worker,” Contact Dermatitis 78, no. 6 (2018): 427–428, 10.1111/cod.12970. 29446105

[62] I. I. Decuyper , B. J. Green , G. L. Sussman , et al., “Occupational Allergies to Cannabis,” Journal of Allergy and Clinical Immunology in Practice 8, no. 10 (2020): 3331–3338, 10.1016/j.jaip.2020.09.003. 33161961 PMC7837257

[63] H. Aksoy , N. Akdeniz , and F. Karakurt , “Prevalence of Type I Allergy to Latex and Type IV Allergy to Rubber Additives in Turkish Healthcare Workers,” Dermatology Practical & Conceptual 13, no. 3 (2023): e2023187, 10.5826/dpc.1303a187. 37557117 PMC10412029

[64] G. M. Liss , G. L. Sussman , K. Deal , et al., “Latex Allergy: Epidemiological Study of 1351 Hospital Workers,” Occupational and Environmental Medicine 54, no. 5 (1997): 335–342, 10.1136/oem.54.5.335. 9196456 PMC1128782

[65] K. J. Kelly and G. Sussman , “Latex Allergy: Where Are we Now and How Did we Get There?,” Journal of Allergy and Clinical Immunology in Practice 5, no. 5 (2017): 1212–1216, 10.1016/j.jaip.2017.05.029. 28888250

[66] C. Ngamchokwathana and N. Chaiear , “Latex Anaphylaxis in Healthcare Worker and the Occupational Health Management Perspective: A Case Report,” SAGE Open Medical Case Reports 11 (2023): 2050313X231179303, 10.1177/2050313X231179303. PMC10265348 37325168

[67] K. Alanko , T. Tuomi , M. Vanhanen , et al., “Occupational IgE‐Mediated Allergy to *Tribolium confusum* (Confused Flour Beetle),” Allergy 55, no. 9 (2000): 879–882, 10.1034/j.1398-9995.2000.00572.x. 11003453

[68] A. L. Lopata , B. Fenemore , M. F. Jeebhay , G. Gade , and P. C. Potter , “Occupational Allergy in Laboratory Workers Caused by the African Migratory Grasshopper Locusta Migratoria,” Allergy 60, no. 2 (2005): 200–205, 10.1111/j.1398-9995.2005.00661.x. 15647041

[69] R. Rauschenberg , A. Bauer , S. Beissert , and P. Spornraft‐Ragaller , “Occupational Immediate‐Type Allergy to Locusts in a Zookeeper,” Journal der Deutschen Dermatologischen Gesellschaft = Journal of the German Society of Dermatology: JDDG 13, no. 2 (2015): 157–158, 10.1111/ddg.12442. 25631136

[70] A. Armentia , R. Alvarez , V. Moreno‐Gonzalez , et al., “Occupational Airborne Contact Urticaria, Anaphylaxis and Asthma in Farmers and Agronomists due to Bruchus Pisorum,” Contact Dermatitis 83, no. 6 (2020): 466–474, 10.1111/cod.13644. 32592184

[71] H. Suojalehto , K. Karvala , S. Ahonen , et al., “3‐(Bromomethyl)‐2‐Chloro‐4‐(Methylsulfonyl)‐Benzoic Acid: A New Cause of Sensitiser Induced Occupational Asthma, Rhinitis and Urticaria,” Occupational and Environmental Medicine 75, no. 4 (2018): 277–282, 10.1136/oemed-2017-104505. 29175989

[72] E. Helaskoski , O. Kuuliala , and K. Aalto‐Korte , “Occupational Contact Urticaria Caused by Cyclic Acid Anhydrides,” Contact Dermatitis 60, no. 4 (2009): 214–221, 10.1111/j.1600-0536.2009.01526.x. 19338590

[73] G. Moscato , E. Galdi , J. Scibilia , et al., “Occupational Asthma, Rhinitis and Urticaria due to Piperacillin Sodium in a Pharmaceutical Worker,” European Respiratory Journal 8, no. 3 (1995): 467–469, 10.1183/09031936.95.08030467. 7789496

[74] E. Helaskoski , H. Suojalehto , H. Virtanen , et al., “Occupational Asthma, Rhinitis, and Contact Urticaria Caused by Oxidative Hair Dyes in Hairdressers,” Annals of Allergy, Asthma & Immunology : Official Publication of the American College of Allergy, Asthma, & Immunology 112, no. 1 (2014): 46–52, 10.1016/j.anai.2013.10.002. 24331393

[75] M. Pesonen , L. Airaksinen , R. Voutilainen , R. Riekki , S. Jungewelter , and K. Suuronen , “Occupational Contact Urticaria and Rhinitis Caused by Immediate Allergy to Palladium Salts,” Contact Dermatitis 71, no. 3 (2014): 176–177, 10.1111/cod.12214. 25155073

[76] T. Estlander , L. Kanerva , O. Tupasela , H. Keskinen , and R. Jolanki , “Immediate and Delayed Allergy to Nickel With Contact Urticaria, Rhinitis, Asthma and Contact Dermatitis,” Clinical and Experimental Allergy : Journal of the British Society for Allergy and Clinical Immunology 23, no. 4 (1993): 306–310, 10.1111/j.1365-2222.1993.tb00327.x. 8319128

[77] M. Hoekstra , S. van der Heide , P. J. Coenraads , and M. L. Schuttelaar , “Anaphylaxis and Severe Systemic Reactions Caused by Skin Contact With Persulfates in Hair‐Bleaching Products,” Contact Dermatitis 66, no. 6 (2012): 317–322, 10.1111/j.1600-0536.2012.02047.x. 22568838

[78] K. Aalto‐Korte and S. Makinen‐Kiljunen , “Specific Immunoglobulin E in Patients With Immediate Persulfate Hypersensitivity,” Contact Dermatitis 49, no. 1 (2003): 22–25. 14641116 10.1111/j.0105-1873.2003.00134.x

[79] L. Kanerva , H. Hyry , R. Jolanki , M. Hytonen , and T. Estlander , “Delayed and Immediate Allergy Caused by Methylhexahydrophthalic Anhydride,” Contact Dermatitis 36, no. 1 (1997): 34–38, 10.1111/j.1600-0536.1997.tb00919.x. 9034685

[80] R. Merget , N. Pham , M. Schmidtke , et al., “Medical Surveillance and Long‐Term Prognosis of Occupational Allergy due to Platinum Salts,” International Archives of Occupational and Environmental Health 90, no. 1 (2017): 73–81, 10.1007/s00420-016-1172-0. 27734174

[81] A. Cristaudo , F. Sera , V. Severino , M. De Rocco , E. Di Lella , and M. Picardo , “Occupational Hypersensitivity to Metal Salts, Including Platinum, in the Secondary Industry,” Allergy 60, no. 2 (2005): 159–164, 10.1111/j.1398-9995.2004.00521.x. 15647035

[82] R. A. Balogun , A. Siracusa , and D. Shusterman , “Occupational Rhinitis and Occupational Asthma: Association or Progression?,” American Journal of Industrial Medicine 61, no. 4 (2018): 293–307, 10.1002/ajim.22819. 29411403

[83] R. Treudler , M. Worm , A. Bauer , et al., “Occupational Anaphylaxis: A Position Paper of the German Society of Allergology and Clinical Immunology (DGAKI),” Allergologie Select 8 (2024): 407–424, 10.5414/alx02543e. 39659712 PMC11629776

[84] H. Süß , S. Dölle‐Bierke , J. Geier , et al., “Contact Urticaria: Frequency, Elicitors and Cofactors in Three Cohorts (Information Network of Departments of Dermatology; Network of Anaphylaxis; and Department of Dermatology, University Hospital Erlangen, Germany),” Contact Dermatitis 81, no. 5 (2019): 341–353, 10.1111/cod.13331. 31173644

[85] V. Ojanen , M. Korhonen , K. Koskela , T. Reho , and R. Sauni , “Predictive Factors Associated With the Utilization of Vocational Rehabilitation for Occupational Diseases in Finland: A Multiregister Analysis,” Journal of Occupational and Environmental Medicine 67, no. 9 (2025): e597–e604, 10.1097/JOM.0000000000003435. 40436625 PMC12379794

