# Supplementary material for: Occupational Contact Urticaria, Protein Contact Dermatitis and Concomitant Airway Diseases in the Finnish Register of Occupational Diseases in 2005–2020: Main Causes and Occupations at Risk
Source: Contact Dermatitis. 2026 Mar 12;95(1):74–85. doi: 10.1111/cod.70137 (PMC13238402; doi:10.1111/cod.70137)
Supplement: Supplementary file 1 — Table S1: Proportion of cases with the same causative exposure for combinations of immediate occupational diseases. [file COD-95-74-s001.pdf]

Table S1. Proportion of cases with the same causative exposure for combinations of immediate occupational diseases.

Disease combination	Same causative exposure<br>n (%)
OR and OA (airway disease only)	192 (95)
OCU/PCD and OR	74 (84)
OCU/PCD and OA	36 (88)
OCU/PCD, OR and OA<br>(all three diagnoses)	56 (86)
OCU/PCD and OA and/or OR<br>(concomitant skin and airway disease (CSAD))	172 (89)

OR, occupational rhinitis; OA, occupational asthma; OCU/PCD, occupational contact urticaria and/or protein contact dermatitis.
