# Supplementary material for: Occupational Contact Urticaria, Protein Contact Dermatitis and Concomitant Airway Diseases in the Finnish Register of Occupational Diseases in 2005–2020: Main Causes and Occupations at Risk
Source: Contact Dermatitis. 2026 Mar 12;95(1):74–85. doi: 10.1111/cod.70137 (PMC13238402; doi:10.1111/cod.70137)
Supplement: Supplementary file 2 — Table S2: ISCO codes and mean workforces for occupations presented in Table 2. Mean workforce calculated as the mean of workforce in years 2010–2020 according to Statistics Finland [13]. [file COD-95-74-s002.pdf]

Table S2. ISCO codes and mean workforces for occupations presented in Table 2. Mean workforce calculated as the mean of workforce in years 2010-2020 according to Statistics Finland [13].

Occupations	ISCO code	Mean workforce 2010-2020
**Farming and animal care:**		
Mixed crop and animal producers	6130	12652
Gardeners, Horticultural and nursery growers	6113	6500
Livestock and dairy producers	6121	20305
Others (incl. Market-oriented skilled agricultural workers, Veterinarians, Veterinary technicians and assistants, Pet groomers and animal care workers)	61, 2250, 3240, 5164	ND
**Food-related:**		
Bakers, pastry-cooks and confectionery makers	7512	2525
Food and related products machine operators	8160	15059
Cooks	5120	40235
Others (incl. Butchers, Fishmongers and related food preparers, Fast food preparers, Kitchen helpers)	7511, 9411, 9412	ND
**Industrial** incl. Pelt dressers, Tanners and fellmongers, Life science technicians (excluding medical), Chemical products plant and machine operators, Metal working machine tool setters and operators, Motor vehicle mechanics and repairers, House builders, Electrical Equipment Installers and Repairers	7535, 3141, 8131, 7223, 7231, 7111, 741	ND
**Miscellaneous:**		
Hairdressers	5141	12808
Shop sales assistants	5223	103700
Others (incl. Chemical and physical science technicians, Biologists, botanists, zoologists and related professionals, Shop keepers, Shop supervisors, Freight handlers, Personal service workers)	3111, 2131, 5221, 5222, 9333, 51	ND
**Occupation changed**		ND
**Occupations without concomitant diseases**		ND
**Total**		2291557
